# Current Oncology Nutrition Care Practice in Southeast Asia: A Scoping Review

**DOI:** 10.3390/nu16101427

**Published:** 2024-05-09

**Authors:** Choirun Nissa, Lauren Hanna, Judy Bauer

**Affiliations:** 1 Department of Nutrition, Dietetics and Food, Monash University, Notting Hill, VIC 3168, Australia; choirun.nissa@monash.edu (C.N.); lauren.hanna@monash.edu (L.H.); 2 Department of Nutrition Science, Faculty of Medicine, Diponegoro University, Jl. Prof. Mr. Sunario, Tembalang, Semarang City 50275, Indonesia

**Keywords:** oncology, cancer, nutritionist, dietitian, nutrition care practice, review

## Abstract

Although evidence-based nutrition care is recommended for patients with cancer, current nutrition care practices provided by nutritionists and dietitians in Southeast Asian countries are not clearly reported. The aim of this scoping review was to describe nutritionists’ and dietitians’ current oncology nutrition care practice within Southeast Asia by identifying access to dietetic services, tools or strategies used in providing care, and barriers and enablers to implementing nutrition care practices. Five databases (Ovid MEDLINE, Global Health, Embase, Cochrane Central Register of Controlled Trials, and Proquest) were searched through structured search strategies, in addition to strategic searching of grey literature. A total of 4261 sources of evidence were retrieved. After full-text screening, 18 studies from Southeast Asian countries met the inclusion criteria and were included in this review. The provision and reporting of nutrition care practices provided by nutritionists and dietitians were limited. Access to dietetic services, including nutritional screening tools and reason to be referred, were varied within studies. Barriers and enablers to nutrition care provision were unique and related to each country’s specific resources and guidelines. In summary, there was varied reporting of nutrition care practices provided to patients with cancer in Southeast Asia and a lack of clarity on the actual standardized processes. Future research is warranted to further explore the barriers and enablers to providing nutrition care by local nutritionists and dietitians in Southeast Asia.

## 1. Introduction

Patients with cancer are highly prone to disease-related malnutrition [1], leading to a lower quality of life [2] and higher mortality [3]. Cancer-associated malnutrition occurs due to multiple disease-specific factors and treatments such as decreased desire to eat, eating difficulties, gastrointestinal motility disorder, decreased capacity to utilize nutrients, or increased energy expenditure [4]. Inadequate food intake may be due to nutrition impact symptoms (NIS) such as poor appetite, nausea, vomiting, taste and smell alteration, mucositis, constipation, and fatigue, which are associated with complications of advanced cancer and anticancer treatment [5,6]. A multi-center study involving 4783 patients with cancer showed that 34% of patients with moderate or suspected malnutrition and 51.8% of patients with severe malnutrition had more than three NIS present [7]. Since patients have different NIS due to their diagnosis and treatment, individualized nutrition care practice with adequate assessment and nutritional intervention should be given in parallel with cancer care in order to manage these NIS and improve nutritional intake [8,9].

As it has been estimated that almost 20% of patients with cancer die from malnutrition rather than the cancer itself [10,11], nutrition plays a pivotal role in cancer care. To prevent nutritional decline, The European Society for Parenteral and Enteral Nutrition (ESPEN) strongly recommends screening and assessment at an early stage to determine appropriate nutrition care [11]. ESPEN also recommends nutritional intervention to increase oral intake in patients with cancer, including dietary advice provided by nutritionists or dietitians [12], as nutritional counseling and adequate supportive care contribute to improved quality of life, satisfaction, and survival [8].

The reported prevalence of malnutrition in patients with cancer in Southeast Asia is varied. Individual studies conducted in Indonesia, Malaysia, and Vietnam reported that 50–85% of patients with cancer were at risk of severe malnutrition [13,14,15,16]. Despite this high risk, not every patient with cancer has access to oncology nutrition care provided by nutritionists and dietitians due to limited staffing resources in Southeast Asian countries [17]. For example, Indonesia has a population of more than 278 million [18], with 30,861 nutritionists and dietitians working in hospitals and community health centers [19], resulting in a ratio of 11 nutritionists and dietitians for every 100,000 of the Indonesian population. This is lower than the 2024 Indonesian Development Plan benchmark of 18 per 100,000 [20]. Compared to the Australian 2017 benchmark data of 15 dietitians per 100,000 population [21], Indonesia’s workforce of nutritionists and dietitians is inadequate to provide nutrition care.

In 2003, the Academy of Nutrition and Dietetics (United States) developed the Nutrition Care Process (NCP) for dietetic professionals, which provides a standardized framework for nutrition care that can be applied to individuals [22]. There are four distinct yet interrelated steps of the NCP: nutrition assessment, nutrition diagnosis, nutrition intervention, and nutrition monitoring/evaluation [22,23]. This framework has been standardized with the use of international terminology to document the process (2008), [24], to the use of terminology in gaining efficacy (2019) [25], and application outside clinical practice (2021) [26]. Ensuring access to nutritionists and dietitians for nutritional assessment, diagnosis of malnutrition, provision of interventions to improve nutrition status, and nutritional monitoring is crucial to quality healthcare [17]. To date, published information on current oncology nutrition care practices provided by nutritionists and dietitians in Southeast Asia is limited.

This scoping review aimed to describe the real-world oncology nutrition care practice of nutritionists and dietitians in Southeast Asia by identifying (a) access to dietetic services, including nutritional screening and dietitian referral, (b) tools or strategies used in providing nutrition care, particularly in nutritional screening, assessment, or referral to a dietitian, and (c) barriers and enablers to implementing nutrition care practices.

## 2. Materials and Methods

The review was conducted per the Preferred Reporting Items for Systematic Review and Meta-Analyses extension for Scoping Reviews (PRISMA-ScR) checklist [27]. The protocol for this scoping review was prospectively registered with the Open Science Framework on 24 November 2023 (https://doi.org/10.17605/OSF.IO/BG8AX) prior to data extraction and analysis.

### 2.1. Search Strategy

Three elements were used to define a clear title and inclusion criteria for the scoping review, in line with guidance from the Joanna Briggs Institute (JBI) [28]. Aspects of ‘Population’ or concept #1 (Dietitians/nutritionists), ‘Concept’ or concept #2 (Southeast Asia), and ‘Context’ or concept #3 (Cancer treatment/oncology) were developed to answer the research question: “What evidence is available regarding current clinical practice of nutritionists and dietitians working in cancer treatment centers in Southeast Asia?”.

The search strategy, including all identified keywords and terms, was adapted for each database and information sources to capture published and unpublished evidence or grey literature. For the published evidence, the search strategy was developed using Ovid MEDLINE 2023 database guide and was guided by an experienced medical librarian.

Five electronic databases were systematically searched to identify relevant publications: MEDLINE (Ovid), Global Health (Ovid), Embase (Ovid), Cochrane Central Register of Controlled Trials (Ovid), and Proquest. Medical subject heading (MeSH) and keywords “nutritionists”, “Southeast Asia”, and “cancer” were combined and used in the database searches. All search strategies were piloted using MEDLINE, and modifications were made to the final search terms. The MEDLINE search strategy is provided in Table 1; search strategies for additional databases are available in Supplementary Materials.

Iterative searches for grey literature were also performed through three distinct searching strategies, which were informed by a study by Godin et al. [29]: (1) grey literature database (ProQuest Dissertation and Theses Global), (2) targeted website (nutrition associations in Southeast Asia), and (3) consultation with contact experts, which is available in Supplementary Materials for further detail. For targeted websites, the searches used the keywords “cancer” or “kanker” (specifically for Indonesian websites).

Database searches were undertaken on 17 August 2023, and the grey literature was searched on 12 September 2023 by two researchers. No restriction on language was applied. Inclusion criteria were articles reporting information regarding the usual nutrition care practices of qualified nutritionists and/or dietitians working in an oncology setting, where these practices were targeted for adult cancer patients (18 or above), in studies conducted in Southeast Asia and published from 2003 until the present. This period was chosen as the NCP framework was published in 2003 [22]. Articles reporting nutrition care practices of nutritionists or dietitians that were combined with other professions or combined with non-cancer patients and could not be isolated and detailed nutrition care practice from the patient’s perspective were excluded.

### 2.2. Study Selection

All references obtained were exported to Endnote 20 [30] to remove duplication. Two researchers independently used Covidence systematic review software (https://www.covidence.org/, accessed on 23 August 2023) to screen the article’s title, abstract, and keywords [31]. If the abstract did not provide sufficient information, the same researcher examined the full-text article along with inclusion criteria for relevance. Any disparity was resolved by consensus. If no agreement was reached, a recommendation from the third researcher was sought.

### 2.3. Data Extraction

Data extraction was conducted using a customized Excel spreadsheet developed through piloting and discussion between researchers. Extracted data included author, study country and year of publication, study design, cancer type, cancer treatment, specification of profession providing nutrition care practice (dietitian/nutritionist), details of nutrition care practice, and other key findings. Information extracted was limited to the relevancy to inclusion criteria.

## 3. Results

### 3.1. Search Result

**Figure 1:**
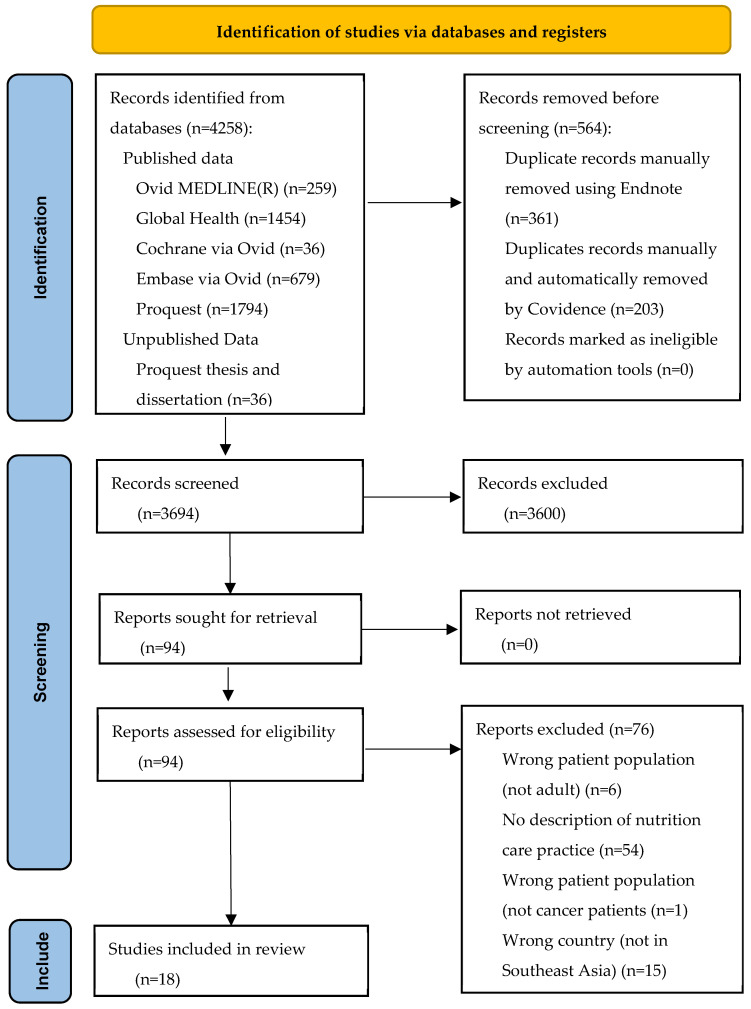
PRISMA flow diagram of study selection.

The PRISMA flow diagram is shown in Figure 1. A total of 4258 studies from six databases and three records from grey literature were retrieved. Following manual removal and automation of 564 duplicates through Endnote and Covidence, 3694 studies underwent title and abstract screening, with 3600 studies deemed irrelevant. Ninety-four studies were left for full-text screening, while 76 were excluded for secondary reasons, as shown in Figure 1. A total of 18 studies were included in this scoping review.

### 3.2. Study Characteristics

Characteristics of the 18 studies are summarized in Table 2 [32,33,34,35,36,37,38,39,40,41,42,43,44,45,46,47,48,49]. Studies were published between 2012–2022 from five of 11 countries (45%) listed in Southeast Asia: Malaysia (*n* = 10, 56%) [32,37,38,39,40,41,44,47,48,49], Vietnam (*n* = 4, 22%) [33,35,36,42], Philippines (*n* = 2, 11%) [45,46], Singapore [34] and Thailand [43] (both *n* = 1, 6%). Half of the studies [32,38,39,40,42,45,46,47,48] were published in 2020 or later, while the remaining studies were published from 2012–2019 [33,34,35,36,37,41,43,44,49]. Nine studies used observational study design, with most (*n* = 7) undertaken as a prospective observational study [35,36,38,41,43,44,46], retrospective [33] or prospective cohort [40]. The remaining studies were audits [34,37], study protocols [32,47], randomized controlled trials (RCT) [48,49], qualitative studies [39,45], and a quasi-experimental study [42].

In 16 studies (89%) [32,33,34,35,36,38,39,40,41,42,43,44,46,47,48,49], participants were patients with cancer, while in two studies (11%), participants were a mix of patients and health professionals; the health professionals were nurses and medical professionals in one study [37], and nurses, nutritionists and dietitians, oncologists, study coordinators, and surgeons in the other study [45].

The cancer type of participants most frequently reported was colorectal cancer (*n* = 3) [43,46,49], followed by breast cancer (*n* = 2) [39,40]. Three studies involved two cancer types [42,47,48]. In comparison, four studies involved cancer of a particular region, i.e., head and neck cancer (*n* = 1) [38], hematological malignancies (*n* = 1) [34], gastrointestinal cancer (*n* = 2) [32,33], while two studies examined newly diagnosed cancer patients, with all cancer types eligible to participate [41,44]. Four studies did not detail the cancer type of patients [35,36,37,45].

Single cancer treatments reported were predominantly surgery (*n* = 6) [32,33,43,45,47,48], chemotherapy (*n* = 2) [42,49] and radiotherapy (*n* = 1) [37] while multiple treatments were reported in two studies [34,38]. Any combination of different therapies experienced by patients, either surgery, chemotherapy, or radiotherapy, was applied in three studies [39,40,46]. In the remaining studies, participants had not yet started treatment [41,44], or the treatment regime was unclear [35,36].

Nutrition care was provided by dietitians in 11 studies [32,33,34,36,37,38,40,44,46,47,49], both nutritionists and dietitians in five studies [39,41,42,43,45], unspecified staff in the nutrition and dietetics department in one study [35], and not stated in one study [48].

### 3.3. Nutrition Screening

Five studies (28%) reported that nutritional screening was conducted at their center, using either the Malnutrition Screening Tool (MST) (Malaysia) [32,37,42,50], the 3-Minute Nutrition Screening (3-MinNS) tool (Singapore) [34,51], or the Nutritional Risk Assessment Tool (Philippines) [45,52]. Screening was performed by nursing staff in two studies (Singapore [34], Malaysia [37], while in one study (Philippines) [45] screening was conducted by either a nurse or a junior resident, with dietitians calculating body mass index (BMI) [45]. One study (Malaysia) reported that nutritional screening was rarely performed and did not state the tool used [44]. In four studies (Malaysia [32,44] Vietnam [35,42]), the staff responsible for conducting nutrition screening was not reported.

**Table 2:** Nutrition care practice information reported in the included studies.

Publication, Study Design, Sample Size	Cancer Treatment	Nutrition Care Provider	Nutrition Screening	Nutrition Assessment	Nutrition Intervention Current Practice	Nutrition Monitoring and Evaluation	Other Key Findings
A’zim et al. [32] Malaysia 2022 Study protocol for a pragmatic randomized control trial.	Gastrointestinal cancer. Major elective gastrointestinal and oncological surgery. No further details on treatment reported.	Dietitian	Malnutrition Screening Tool	Scored PG-SGA and triage based on score: 0–1: no intervention 2–3: health education 4–8: dietetic intervention ≥9: nutrition support.	Nutritional advice based on a guideline focused on the treatment of symptoms (nausea, vomiting, loss of appetite, diarrhea) provided by medical professionals or nurses. Patients with moderate and severe malnutrition referred to a dietitian for nutrition counseling. No routine pre/postoperative ONS.	Preoperative nutritional status monitored via phone.	-
Loan et al. [33] Vietnam 2018 Retrospective cohort study (*n* = 459)	Colorectal (*n* = 291), gastric (*n* = 149), esophageal (*n* = 19) cancer. Major curative surgery. Patients having chemo- or radiotherapy excluded.	Dietitian	-	Preoperative malnutrition defined as underweight classification or hypoalbuminemia within 30 days pre-surgery (via retrospective medical record review).	No preoperative hospital nutrition support due to routine overcrowding (even when clinically indicated). No details on post-operative nutritional support	-	Lack of license system for dietitians. Health insurance did not cover the cost of nutrition treatment. Poor people cannot afford enteral formula.
Chen et al. [34] Singapore 2012 Pre- and post-implementation audit (*n* = 24)	Oncological and hematological malignancy. Chemotherapy. No further details provided.	Dietitian	No nutritional screening within 24 h of admission. 3-MinNS was introduced to nurses by dietitians and incorporated into the nursing admission assessment form. Language barrier on nutritional screening solved with a colleague’s help.	3-MinNS component (weight, food intake—meals, oral supplement, or tube feeding- and muscle wastage) assessed to determine its score.	-	-	No dietitian referral initiated on admission by medical professionals. Online system used during admission for referral to dietitians by medical professionals and nurses.
Cuong et al. [35] Vietnam 2018 Prospective observational Study (total *n* = 883, *n* = 44 cancer)	No cancer type or treatment type reported.	Nutrition and dietetics department staff.	Policies and guidelines to identify and manage hospital malnutrition were in the preliminary stage of development and implementation,	-	No hospital food provided as routine care. Food purchased from hospital canteen, outside hospital, or brought from home.	-	-
Hanna et al. [36] Vietnam 2016 Prospective observational study (total *n* = 372, *n* = 8 cancer)	No cancer type reported. Various treatments.	Dietitians	-	Subjective Global Assessment. Weight measured by calibrated body composition analyzer, (Thong Nat Hospital) or self-reported (Can Tho Hospital)	Thong Nhat Hospital: nutrition department and food service providing meals. Can Tho Hospital: No food service providing meals.	-	Lack of hospital food service infrastructure, less developed nutrition services, and a lack of health insurance funding for hospital food.
Han et al. [37] Malaysia 2018 Clinical audit (*n* = 739 (patients), *n* = 18 (nurses), *n* = 15 (medical professional)).	No cancer type or treatment type reported.	Dietitians	MST completed by nurse at outpatient appointment (new or follow up visit). Nurse completed a bilingual (English-Malay) electronic MST questionnaire. Nurse informed the attending medical professional to complete an EMR dietitian referral.	-	-	-	Dietitian referrals based on MST scores or on medical professionals’ clinical judgement. Referral policy changed from medical professionals to auto-referral, where nurses directly schedule dietitian appointments in an electronic system).
Kay et al. [38] Malaysia 2020 Prospective observational study (*n* = 50)	Head and neck cancer. Prior to radiotherapy and concurrent chemotherapy and radiotherapy.	Dietitians	-	Patients only referred to dietitians when having inadequate dietary intake during treatment. PG-SGA and one day 24 h recall used. No further information on routine practice.	Only 32.1% of malnourished patients referred to a dietitian on admission for nutrition management. No information on nutritional intervention provided.	-	A total of 67.9% of malnourished patients had no dietitian referral. No information on how dietitian referral conducted.
Kiew et al. [39] Malaysia 2022 Qualitative study (interviews) (*n* = 20)	Breast cancer. Post-primary treatment (surgery, chemotherapy, hormonal therapy, radiotherapy).	Dietitians and Nutritionists	-	-	Three of 20 patients met with the dietitian because of other medical conditions requiring dietary counseling (e.g., a healthy eating recommendation). One patient reported that different recommendations were provided by medical professionals and nutritionists.	-	-
Kiew et al. [40] Malaysia 2022 Prospective cohort study (*n* = 112)	Breast cancer. 9–15 months post-treatment (surgery, chemotherapy, hormonal therapy, radiotherapy).	Dietitians	-	-	Lack of dietary guidance from dietitians or clinicians post-active treatment.	-	-
Krishnasamy et al. [41] Malaysia 2017 Prospective observational study (*n* = 132)	Gastrointestinal (*n* = 44), breast (*n* = 20), sarcoma (*n* = 20), head and neck (*n* = 14), lung (*n* = 15), hematologic (*n* = 7), thyroid (*n* = 5), genitourinary (*n* = 4), and gynecologic (*n* = 3) cancers. No cancer treatment prior to study	Dietitians and nutritionists.	-	Subjective Global Assessment	-	-	Severely malnourished patients commonly referred to dietitians or nutritionists. No further information reported on how dietitian referral was conducted.
Huong et al. [42] Vietnam 2021 Quasi-experimental study (*n* = 120)	Stomach and colon cancer. Chemotherapy.	Dietitians and nutritionists	MST within 24 h of admission.	Nutritional status using anthropometric measurement (weight, BMI, % weight loss, MUAC, muscle mass, fat mass), PG-SGA, and laboratory measurement (albumin, prealbumin, total protein).	Nutrition counseling. No further details available.	-	Limited guidelines in nutritional support for patients with cancer in Vietnam.
Lohsiriwat V. [43] Thailand 2014 Prospective observational study (*n* = 149)	Colorectal adenocarcinoma. Elective surgical resection within the ERAS program.	Dietitians or nutritionists	-	Subjective Global Assessment on Admission.	No particular nutrition support protocol for malnourished patients within the modified ERAS program. No preoperative enteral fluid and carbohydrate loading as recommended in the ERAS pathway included in the modified ERAS program. ONS provided during early postoperative period for patients with inadequate food intake and no dietitian input.	-	-
Menon et al. [44] Malaysia 2014 Prospective observational study (*n* = 70)	Digestive organs (*n* = 22), breast (*n* = 13), thyroid (*n* = 9), respiratory system (*n* = 8), genitourinary (*n* = 6), and others, i.e., bone, cervix, head and neck, blood, and bladder cancers (*n* = 12). Newly diagnosed, pre-treatment.	Dietitians	Nutrition screening rarely performed.	-	-	Recommendations for evaluation of nutritional status using standardized protocol existed but were neglected in routine practice.	Terminally ill patients with severe malnourished cancer often referred to dietitians.
Sowerbutts et al. [45] Philippines 2022 Qualitative semi-structured interviews and focus groups (*n* = 2 (nurses), 10 (dietitians or nutritionists), 3 (oncologists), 1 (study coordinator), 8 (surgeons), 10 (patients)). Not all health professionals came from the Philippines.	Healthcare professionals and patients on a surgical cancer ward. No information on cancer type and treatment provided.	Dietitians or Nutritionists	A junior resident or nurse conducted screening using a Nutrition Risk Assessment. Staff missed performing screening due to the old chart not being included in the EMR. Nurses trained by dietitians in the use of screening tools.	The initial assessment occurred with outpatients. Nutritional Risk Assessment combined subjective with objective methods. Patients routinely weighed.	Nutritional advice provided to outpatients by nutritionists. Parenteral nutrition occasionally used. Patients received meals daily, however, purchased additional food as a supplement. Supply of readymade supplements occasionally ran out. ONS inconsistently supplied to or consumed by outpatients.	Patients did not attend the healthcare facility for the sole purpose of nutritional assessment or monitoring.	Patients referred to a dietitian as an outpatient.
Velasco et al. [46] Philippine 2022 Prospective observational study (*n* = 292)	Colorectal cancer. Active treatment: chemotherapy, pre- or post-surgery, or radiotherapy)	Dietitians	-	Formal nutrition assessment not performed in the Philippine General Hospital-Cancer Institute.	-	-	Only 17% of patients referred by attending medical professionals to the dietary service for counseling.
Wong et al. [47] Malaysia 2021 Study Protocol for an open labeled randomized controlled trial.	Breast and colorectal cancer. Elective surgery.	Dietitians	-		Preoperative: Patients received nutrition counseling and a meal plan from a dietitian. Postoperative: the patients received ONS (milk powder drink) until discharged.	-	Milk-based ONS provided postoperatively: 4 leveled scoops of powder (55 g) into 210 mL of lukewarm water. Patients required to consume three servings of ONS per day (750 kcal and 33 g protein per day).
Wong et al. [48] Malaysia 2022 Open-label, multi-arm, parallel-group randomized controlled trial (*n* = 91)	Breast and colorectal cancer. Elective surgery.	Not reported	-	-	Patients received usual diet preoperatively and provided with ONS postoperatively (three servings per day) until discharge.	-	-
Abu Zaid et al. [49] Malaysia 2016 Open-label randomized controlled trial (*n* = 42)	Colorectal cancer. Chemotherapy	Oncologist or nurse, and service dietitian	-	-	Oncologists or nurses gave guideline-based general nutritional advice verbally, visually, and written, focused on symptom treatment. Malnourished patients referred to a service dietitian for dietary counseling.	-	-

3-MinNS, 3-Minutes Nutrition Screening [51]; BMI, Body Mass Index; EMR, Electronic Medical Record; ERAS, Enhanced Recovery After Surgery [53]; MST, Malnutrition Screening Tool [50]; MUAC, Mid-Upper Arm Circumference; ONS, Oral Nutritional Supplement; PG-SGA, Patients Generated-Subjective Global Assessment [54].

Two studies (Singapore [34], Malaysia [37]) reported that nurses conducted online electronic nutrition screening. One study in the Philippines reported a system used for nutrition screening but did not specify whether it was electronic or manual [45]. A Malaysian study described the process of nutrition screening in detail [37]: outpatients were assigned to a screening room by a nurse where patients were weighed and interviewed regarding weight history and appetite changes, and the MST was completed in the electronic medical records (EMR). Following screening, a medical professional was informed to complete a dietitian referral in the EMR [37].

### 3.4. Dietitian Referral

There was variation in the reported reasons for patients being referred to a dietitian. A Singaporean study clearly stated the rationale for dietitian referral, which was scoring of three or more on the nutritional screening tool or being at risk of malnutrition based on the hospital policy [34]. Within the three Malaysian studies [38,41,44], there was inconsistency regarding the reasons for the dietitian referral. The first study reported that the most common dietitian referrals were for severely malnourished patients [41], while the second study stated that only terminally ill patients with severe malnutrition were referred to a dietitian [44]. The third study reported that patients were seldom referred to the dietitian when diagnosed, with referral occurring only when their dietary intake was inadequate during radiotherapy. However, the method for determining dietary adequacy was not described [38].

### 3.5. Assessment Practices

Nutrition screening and assessment practices were described in ten studies [32,33,34,36,38,41,42,43,45,46]. Two studies described the use of the 3-MinNS (Singapore [34,51]) and the Nutritional Risk Assessment (Philippines [45,52]) where they serve both aims: as screening and assessment tools. Six other studies reported that nutritional status was assessed using the Subjective Global Assessment (SGA) [55] (Vietnam [36], Malaysia [41], Thailand [43]), Patient Generated-Subjective Global Assessment (PG-SGA) [54] (Malaysia [32,38], Vietnam [42]). Dietary assessment was highlighted in one study (Malaysia), which reported that patients had inadequate dietary intake during radiotherapy with no detail on the assessment performed [38], while a study in the Philippines reported that nutrition assessment was not routinely performed [46]. Another study (Philippines) described the first outpatient assessment using a Nutritional Risk Assessment tool. Medical professionals asked questions about weight loss, including whether clothes were loose, oral intake, and appetite, before assessing the patient’s weight and height to calculate BMI [45].

A Vietnamese study [33] reported that preoperative malnutrition was assessed retrospectively, either using BMI (with an underweight category according to The International Classification) [56] or hypoalbuminemia. In contrast, another Vietnamese study highlighted the difference between two hospitals’ assessment of weight, one using a calibrated reliable scale and the other relying on patient-reported data [36].

### 3.6. Intervention Practices

Nutrition intervention practices were described in 11 studies (61%) [32,34,35,36,38,39,43,45,47,48,49], including nutrition counseling [32,39,47,49], provision of meal plans [35,36,47], enteral feeding [34], parenteral nutrition [45], and oral nutrition supplements (ONS) [32,34,43,45,47,48]. A Malaysian study reported that only 32% of patients with cancer received nutrition intervention but did not describe how the nutrition intervention was performed [38]. One modified enhanced recovery after surgery (ERAS) study (Thailand) reported the prescription of ONS in the early postoperative period for patients with inadequate food intake but did not have a preoperative nutrition support protocol [43]. Two Malaysian studies in patients with breast and colorectal cancers reported the use of ONS postoperatively until discharge [47,48]. One study (Philippines) reported that ONS was not consistently available or consumed by patients [45], while a Singaporean study reported having a protocol for ONS or tube feeding along with meals according to nutrition risk screening [34]. A Malaysian study stated that ONS was not provided [32].

The provision of meals varied between studies, with two studies stating that meals were not provided to hospital patients (Vietnam) [35,36]. One study (Philippines) reported that meals were provided, although some patients purchased food to supplement their intake due to poor appetite and dislike of the food; this study also reported that parenteral nutrition was occasionally used [45].

### 3.7. Nutrition Monitoring and Evaluation Practices

Three studies (17%) reported on nutritional status monitoring and evaluation practices [32,44,45]. A Malaysian study reported that peri-operative nutritional status was monitored daily using phone calls, including when complications occurred [32]. In contrast, another Malaysian study reported that in routine practice, evaluation of nutritional status was not undertaken [44]. Patients in the Philippines reported being unwilling to attend the healthcare facility for the sole purpose of nutrition assessment or monitoring [45].

### 3.8. Barriers to Accessing and Providing Nutritional Care

Twenty-one barriers were described across five different areas of nutrition care provision: nutritional screening (six barriers) [34,37,45], dietitian referral (five barriers) [34,37,42], nutrition intervention (four barriers) [33,35,36], nutrition evaluation (three barriers) [42,45], and in the provision of nutritional care in general (three barriers) [35,40,42,43]. Barriers to completion of nutrition screening were acknowledged in three studies (17%) [34,37,45]: the complexity of the nutrition screening tool, language barriers between nurses and patients, and mathematical errors in completing the screening score were barriers identified in Singapore [34]. To overcome this, dietitians introduced a validated simplified screening tool [34]. Time limitation was a barrier in a Malaysian study, with the root causes being the inadequacy of staff assigned, lack of familiarity with the tool during interviews for translation, and delay in EMR documentation [37]. Strategies to facilitate the screening process were reported in this study, such as developing a bilingual MST form (Malay and English), changing the screening procedure from staff-administered face-to-face interviews to patient-administered screening, and collecting MST forms for charting in the EMR [37]. Another barrier to nutritional screening, highlighted in a Philippines study, was the lack of clear responsibility for completing the screening tool and no automated EMR prompting staff to conduct nutritional screening [45].

A Vietnamese study reported barriers to referring to dietitians: a lack of clear guidelines, knowledge or training provided, and time constraints [42]. Other barriers acknowledged were nurses not being empowered to initiate dietitian referrals (Singapore) [34] and lack of awareness of the appropriate referral procedure by medical professionals (Malaysia) [37]. Although around 70% of patients with head and neck cancer were reported as malnourished in a Malaysian study, dietitian referrals were not completed due to a lack of nutrition screening early in admission [38].

Several studies stated that a lack of clear guidelines or protocols hindered nutrition care provision. In Vietnam, policies and procedures for screening were in a preliminary stage of development [35], and a lack of clear nutritional care guidelines [42] was reported. The absence of dietary guidelines after completing active treatment (Malaysia) [40] and no nutrition support protocol during the perioperative period (Thailand) [43] were also highlighted as barriers. Similar barriers to accessing dietitians also occurred in performing nutritional care, i.e., limited knowledge or training and time limitation to implement nutritional assessment, intervention, and follow-up (Vietnam) [42]. Furthermore, barriers were highlighted in providing nutrition intervention in two other Vietnamese studies, i.e., routine hospital overcrowding [33], lack of a license system for dietitians [33], inadequate hospital food service infrastructure [36], and health insurance funding [33,36]. Nutritional care provisions for malnourished inpatients were limited in Vietnam [35], with no meals provided to patients in one hospital [36].

Two studies (Philippines) reported that barriers to nutritional evaluation were the long distance to travel and long waiting times [45], and a study in Vietnam described limited time to implement follow-up of nutrition status monitoring [42].

### 3.9. Enablers in Accessing and Providing Nutritional Care

Four main enablers were noted in four studies (22%) related to protocol/policy [34,44], intra-and interdisciplinary collaboration [34,37], training [34,45], and the use of scheduled dietitian clinics [37].

Protocols/policies were described as enablers in two studies. A Malaysian study reported the availability of protocols for the provision of nutrition evaluation [44], while a Singaporean study stated the change of policy from a referral by medical professionals to an automated online referral by nurses increased dietetic services [34].

Other enablers related to collaboration within teamwork were reported in two studies. A multidisciplinary collaboration was noted between nutritionists/dietitians and clinicians (nurses and medical professionals), particularly in performing screening and dietitian referrals (Singapore [34], Malaysia [37]). Nurse teamwork assisted colleagues in overcoming language barriers or calculating the nutritional screening score through constant reinforcement [34]. A Malaysian clinical audit reported that the role of nurses was pivotal in the identification of patients at risk of malnutrition and their referral to dietitians, as the medical professionals reported they were unaware that the MST existed, outpatient screening was mandatory, and unaware of the dietitian referral procedure [37].

Program and training were reported as enablers, particularly in performing nutritional screening. A pre- and post-audit in Singapore revealed an increase in the proportion of patients screened upon admission using a validated screening tool and the proportion of dietitian referrals, following a program by dietitians to educate and empower nursing staff to conduct screening and complete referrals [34]. Two studies (Singapore [34], Philippines [45]) reported that the dietitian provided training to nursing staff regarding the use of screening tools. Additionally, a Malaysian study [37] described the implementation of a weekly dietitian oncology clinic increased patient access to dietitians.

## 4. Discussion

This paper is the first scoping review to examine nutrition care practices provided by nutritionists and dietitians to adult patients with cancer in Southeast Asia. The main findings of this study indicate limited reporting of oncology nutrition care provided by dietitians or nutritionists and high variation in nutrition care practice within Southeast Asia. Most of the published research detailed nutrition intervention practices and several studies highlighted the nurse’s role as the main collaborator in ensuring patients’ access to nutrition care.

This scoping review demonstrates that there is a lack of clarity in reporting on standard nutrition care practices in Southeast Asian countries. Although no study in this review reported all four steps of the NCP, studies described two steps, i.e., assessment-intervention [42,43], three steps, i.e., assessment-intervention-evaluation [45], or how referrals for nutrition care were facilitated by nutritional screening [34,44]. The Academy of Nutrition and Dietetics’ strategic plan describes high-quality nutrition care as doing the right thing at the right time, in the right way, for the right person, and achieving the best possible result [22]. Early nutrition care for patients on admission is proven to be associated with improved outcomes during their cancer journey [1]. To optimize health service and patient-related outcomes, all steps of the NCP need to be implemented [22].

The four studies undertaken in Vietnam reported unique nutrition care practices compared to other Southeast Asian countries [32,35,36,42]: most hospitals did not provide meals as part of standard nutrition care due to overcrowding [33], lack of food service infrastructure [36] and limited guidelines for nutritional support [42]. Health insurance in Vietnam does not include the cost of nutrition treatment, making access to hospital meals and ONS for patients difficult [33,36]. Therefore, financial support is required to increase the nutrition and dietetic workforce, ensure adequate training regarding nutrition care, increase the expertise of medical staff, establish food service infrastructure, and develop Vietnamese-based protocols for managing malnutrition using a food-first approach to integrate meal provision into health care, in order to optimize nutrition interventions in Vietnam [35].

Inconsistent and limited use of nutritional support during both preoperative and postoperative care was reported. Perioperative nutritional support included within an ERAS pathway has proven to achieve better surgical results: starting with a preoperative nutritional assessment, avoiding fasting prior to surgery, and correcting malnutrition prior to surgery is beneficial in maintaining nutritional status, enhancing recovery, and minimizing surgical stress and complications [57]. Nutritional support in the postoperative period may reduce the need for intensive care, length of hospital stay, healthcare costs, and mortality [12,57,58]. Only two Malaysian studies [47,48] reported using perioperative ONS. In contrast, a Vietnamese study reported the absence of nutritional support prior to surgery with no detail regarding nutrition intervention performed in the post-surgical period, which resulted in a higher risk of postoperative malnutrition and length of stay [33]. This study recommended strategies to address this issue, i.e., a coalition of hospitals where certain district hospitals provide support for preoperative and postoperative nutrition management of malnourished patients to prevent complications post-surgery [33].

Dietitians and nutritionists were reported as the providers of nutrition care in all studies, with three studies reporting that nutrition care was provided by dietitians in collaboration with other health professionals [32,39,49]. A resolution of the Asian Forum of Dietetic Professionals (AFDP) agreed that the definition of dietitian is based upon that of the respective country/area [59]. For example, the Malaysian Dietitian Association defines a dietitian as a trained professional who is qualified to conduct nutrition assessment, nutrition diagnosis, and nutrition intervention to meet the needs of the individual based on the medical problem [60]; in contrast, nutritionists focus on promoting nutritional well-being to prevent nutrition-related diseases in healthy individuals, communities, or individuals at risk of nutritional diseases/disorders [61,62]. A clear definition and scope were described through an agreement prior to implementing the Allied Health Professions Act (Act 774) through the Nutrition Society of Malaysia, where dietitians focus more on providing Medical Nutrition Therapy to patients. This agreement and the Allied Health Professions Act (Act 774) prevent overlapping between the two professions [62].

Limited time [37,42,45] and lack of clear guidelines [40,42,43] was reported in nearly 20% of the studies and appears to be the two most common barriers to practicing nutrition care in Southeast Asia. This may be due to limited staff, unstandardized roles, and lack of a licensing system for dietitians [33], leading to a high workload that may contribute to their inability to meet the nutrition needs of the patients, triggering staff turnover [63]. Even though workload-related stress as a result of high work demand is also experienced by dietitians in Australia, job satisfaction, i.e., convenience, geographic location to home, supportive leave arrangement, reasonable pay rates, and job flexibility outweigh the drawbacks and enhanced retention of dietitians [64].

Collaboration between health professionals and dietitians has been highlighted as an essential enabler, particularly in nutrition screening and dietitian referral, functioning as the main pathway for patients to access nutrition care [65]. Nurses and medical professionals play a key role in providing dietitian referrals for patients with cancer in Southeast Asia. Patients with cancer value when their usual medical professionals are involved in providing referrals [66]. A Malaysian study described a lack of awareness among medical professionals of their role in completing dietitian referrals after nursing staff had completed malnutrition screening, which may be due to limited formal nutrition education received during training [37] and not being familiar with hospital nutrition care policies. Nurses are often the first healthcare professionals in the multidisciplinary team (MDT) to encounter patients [34] and are responsible for implementing dietitian-led recommendations. To prevent late access to nutrition care, a nursing team in a Singaporean study implemented a strategy that empowered nurses to directly refer malnourished patients to a dietitian [34]. A recent scoping review revealed that best practice NCP implementations involving MDT were facilitated by adequate and sustainable training, effective communication, and information-sharing, with the support of clinical leadership and management, which leads to a collaborative working environment. The outcomes identified in this review were improved nutritional status, staff satisfaction, and overall quality of service for the patients [67].

Strengths of this study include that the review protocol was prospectively registered, and the study followed the PRISMA-ScR framework to ensure that the selection of included studies resulted from a structured method. Multiple reviewers also reduced subjective interpretation, making the data more reliable. The use of the Academy of Nutrition and Dietetics framework to classify nutrition care practices into each of the four steps of the NCP provided a consistent interpretation of available current practices in each country. An additional strength of this study was the use of English and Bahasa in the search strategies, providing a wider range of results. Limitations of the study include not expanding the search to other Southeast Asian languages; therefore, relevant evidence might have been excluded. Additionally, as this was a scoping review, a quality assessment was not undertaken to determine the risk of bias.

## 5. Conclusions

Current oncology nutrition care practices in Southeast Asia were varied and lacked clarity in reporting actual standardized processes. Barriers and enablers were considered unique and country-based due to the specific resources and guidelines available in each setting. It is recommended that future oncology nutrition studies in Southeast Asia provide further detail on each of the steps of the NCP undertaken as part of current practice. Additional research is warranted to further investigate the barriers and enablers to the provision of evidence-based oncology nutrition care in Southeast Asian hospitals.

## Figures and Tables

**Table 1:** MEDLINE search strategy.

Concept #1: Dietitian/Nutritionist	Concept #2: Southeast Asia	Concept #3: Cancer Treatment/Oncology
nutritionists/OR dietetics/OR dietary services/OR nutrition*.mp. OR (dietitian* or dietician* or dietetic*).mp.	exp Asia, Southeastern/OR Asia, southeastern/OR Borneo/OR Brunei/OR Cambodia/OR Indochina/OR Indonesia/OR Laos/OR Malaysia/OR Mekong Valley/OR Myanmar/OR Philippines/OR Singapore/OR Thailand/OR Timor-leste/OR Vietnam/OR (Southeast* Asia OR South-east* Asia* OR Borneo* OR Brunei OR Cambodia* OR Indochina* OR Indo-chin* OR Indonesia* OR Lao* OR Malaysia* OR Mekong Valley OR Myanmar OR Philippine* OR Singapore* OR Thai* Timor-Leste OR Vietnam*).mp OR (Southeast* Asia OR South-east* Asia* OR Borneo* OR Brunei OR Cambodia* OR Indochin* OR Indo-chin* or Indonesia* or Lao* or Malaysia* or Mekong Valley or Myanmar or Philippin* or Singapor* or Thai* Timor-Leste or Vietnam*).cp.	medical oncology/OR radiation oncology/OR surgical oncology/OR cancer care facilities/OR oncology service, hospital/OR exp neoplasms/OR cancer*.mp. OR oncolog*.mp. OR neoplasm*.mp. OR kanker.mp

The ‘*’ indicates truncation of a search term which is standard in systematic database searching.

## Supplementary Materials

The following supporting information can be downloaded at https://www.mdpi.com/article/10.3390/nu16101427/s1. Table S1: Documentation of search strategies of published database; Table S2: Documentation of search strategy for grey literature database; Table S3: Documentation of search strategy for grey literature: targeted website (nutrition association in Southeast Asia); Table S4: Documentation of search strategy for grey literature: contact knowledge expert.
