# Supplementary material for: Current Oncology Nutrition Care Practice in Southeast Asia: A Scoping Review
Source: Nutrients. 2024 May 9;16(10):1427. doi: 10.3390/nu16101427 (PMC11123819; doi:10.3390/nu16101427)
Supplement: Supplementary file 1 [file nutrients-16-01427-s001.zip › nutrients-2971811-supplementary.pdf]

Supplementary Materials

Table S1. Documentation of search strategies of published database

Date	Database name, URL	Search strategies/words searched	# of items retrieved	# of items screened (uploaded to Endnote)
17/Aug/2023	OVID MEDLINE(R) 1946 TO AUGUST 15, 2023 https://ovidsp.dc1.ovid.com/ovid-new-a/ovidweb.cgi?&S=KHICFPHOEHACJMCNKPJJNHOIIKMAAA00&DB+List=1&Cancel=1	#1 Nutritionists/<br>#2 Dietetics/<br>#3 Dietary Services/<br>#4 nutrition*.mp.<br>#5 (dietitian* or dietician* or dietetic*).mp.<br>#6 or/1-5<br>#7 exp Asia, Southeastern/<br>#8 Asia, Southeastern/ or Borneo/ or Brunei/ or Cambodia/ or Indochina/ or Indonesia/ or Laos/ or Malaysia/ or Mekong Valley/ or Myanmar/ or Philippines/ or Singapore/ or Thailand/ or Timor-Leste/ or Vietnam/<br>#9 (Southeast* Asia or South-east* Asia* or Borneo* or Brunei or Cambodia* or Indochin* or Indo-chin* or Indonesia* or Lao* or Malaysia* or Mekong Valley or Myanmar or Philippin* or Singapor* or Thai* Timor-Leste or Vietnam*).mp<br>#10 (Southeast* Asia or South-east* Asia* or Borneo* or Brunei or Cambodia* or Indochin* or Indo-chin* or Indonesia* or Lao* or Malaysia* or Mekong Valley or Myanmar or Philippin* or Singapor* or Thai* Timor-Leste or Vietnam*).cp.<br>#11 or/7-10<br>#12 medical oncology/ or radiation oncology/ or surgical oncology/<br>#13 Cancer Care Facilities/<br>#14 Oncology Service, Hospital/<br>#15 exp Neoplasms/<br>#16 cancer*.mp.<br>#17 oncolog*.mp.<br>#18 neoplasm*.mp.<br>#19 kanker.mp.<br>#20 or/12-19<br>#21 6 and 11 and 20	259	259
17/Aug/2023	GLOBAL HEALTH 1910 TO 2023 WEEK 32 https://ovidsp.dc1.ovid.com/ovid-new-a/ovidweb.cgi?&S=KHICFPHOEHACJMCNKPJJNHOIIKMAAA00&New	#1 dietitians/ or dietetics/<br>#2 nutritionists/<br>#3 nutrition*.mp.<br>#4 (dietitian* or dietician* or dietetic*).mp<br>#5 or/1-4<br>#6 exp south east asia/	1454	1454

Date	Database name, URL	Search strategies/words searched	# of items retrieved	# of items screened (uploaded to Endnote)
	+Database=Single%7c 19	#7 (Southeast* Asia or South-east* Asia* or Borneo* or Brunei or Cambodia* or Indochin* or Indo-chin* or Indonesia* or Lao* or Malaysia* or Mekong Valley or Myanmar or Philippin* or Singapor* or Thai* Timor-Leste or Vietnam*).mp.<br>#8 (Southeast* Asia or South-east* Asia* or Borneo* or Brunei or Cambodia* or Indochin* or Indo-chin* or Indonesia* or Lao* or Malaysia* or Mekong Valley or Myanmar or Philippin* or Singapor* or Thai* Timor-Leste or Vietnam*).cp.gl.<br>#9 or/6-8<br>#10 exp neoplasms/<br>#11 cancer/<br>#12 cancer*.mp.<br>#13 oncolog*.mp.<br>#14 neoplasm*.mp.<br>#15 kanker.mp.<br>#16 or/10-15<br>#17 5 and 9 and 16		
17/Aug/2023	EBM REVIEWS- COCHRANE CENTRAL REGISTER OF CONTROLLED TRIALS JULY 2023 https://ovidsp.dc1.ovid.com/ovid-new-a/ovidweb.cgi?&S=KHICFPHOEHACJMCNKPJJNHOIIKMAAA00&New+Database=Single%7c6	#1 Nutritionists/<br>#2 dietetics/<br>#3 Dietary Services/<br>#4 nutrition*.mp.<br>#5 (dietitian* or dietician* or dietetic*).mp.<br>#6 or/1-5<br>#7 exp Asia, Southeastern/<br>#8 Asia, Southeastern/ or Borneo/ or Brunei/ or Cambodia/ or Indochina/ or Indonesia/ or laos/ or Malaysia/ or mekong valley/ or Myanmar/ or Philippines/ or Singapore/ or Thailand/ or timor-leste/ or Vietnam/<br>#9 (Southeast* Asia or South-east* Asia* or Borneo* or Brunei or Cambodia* or Indochin* or Indo-chin* or Indonesia* or Lao* or Malaysia* or Mekong Valley or Myanmar or Philippin* or Singapor* or Thai* Timor-Leste or Vietnam*).mp.<br>#10 (Southeast* Asia or South-east* Asia* or Borneo* or Brunei or Cambodia* or Indochin* or Indo-chin* or Indonesia* or Lao* or Malaysia* or Mekong Valley or Myanmar or Philippin* or Singapor* or Thai* Timor-Leste or Vietnam*).cp<br>#11 or/7-10	36	36

Date	Database name, URL	Search strategies/words searched	# of items retrieved	# of items screened (uploaded to Endnote)
		#12 medical oncology/ or radiation oncology/ or surgical oncology/<br>#13 Cancer Care Facilities/<br>#14 Oncology Service, Hospital/<br>#15 exp Neoplasms/<br>#16 cancer*.mp.<br>#17 oncolog*.mp.<br>#18 neoplasm*.mp.<br>#19 kanker.mp.<br>#20 or/12-19<br>#21 6 and 11 and 20		
17/Aug/2023	EMBASE CLASSIC+EMBASE 1947 TO 2023 AUGUST 15 https://ovidsp.dc1.ovid.com/ovid-new-a/ovidweb.cgi?&S=KHICFPHOEHACJMCNKPJJNHOIIKMAAA00&New+Database=Single%7c17	#1 Dietitian/<br>#2 Dietetics/<br>#3 Dietary Service/<br>#4 nutrition*.mp.<br>#5 (dietitian* or dietician* or dietetic*).mp<br>#6 or/1-5<br>#7 exp Southeast Asia/<br>#8 (Southeast* Asia or South-east* Asia* or Borneo* or Brunei or Cambodia* or Indochin* or Indo-chin* or Indonesia* or Lao* or Malaysia* or Mekong Valley or Myanmar or Philippin* or Singapor* or Thai* Timor-Leste or Vietnam*).mp.<br>#9 (Southeast* Asia or South-east* Asia* or Borneo* or Brunei or Cambodia* or Indochin* or Indo-chin* or Indonesia* or Lao* or Malaysia* or Mekong Valley or Myanmar or Philippin* or Singapor* or Thai* Timor-Leste or Vietnam*).cp.<br>#10 or/7-9<br>#11 medical oncology/<br>#12 exp Neoplasms/<br>#13 malignant neoplasm/<br>#14 cancer center/<br>#15 cancer*.mp.<br>#16 neoplasm*.mp.<br>#17 kanker.mp.<br>#18 oncology.mp.<br>#19 or/11-18<br>#20 6 and 10 and 19	679	679
17/Aug/2023	PROQUEST https://www.proquest.com/advanced?accountid=12528	#1 dietitians (MeSH) OR nutritionist (MeSH) OR nutrition (MeSH)<br>#2 dietitian* OR dietician* OR dietetic* OR nutrition*<br>#3 OR/1-2<br>#4 Asian people (MeSH)	1794	1794

Date	Database name, URL	Search strategies/words searched	# of items retrieved	# of items screened (uploaded to Endnote)
		#5 Southeast* Asia* OR South-east* Asia* OR Borneo* OR Brunei OR Cambodia* OR Indochin* OR Indo-chin* OR Indonesia* OR Lao* OR Malaysia* OR Mekong Valley OR Myanmar OR Philippin* OR Singapor* OR Thai* OR Timor-Leste OR Vietnam*<br>#6 OR/4-5<br>#7 Oncology (MeSH) OR Cancer therapies (MeSH) OR Tumors (MeSH) OR Cancer (MeSH) OR Cancer surgeries (MeSH) OR radiation therapy (MeSH) OR chemotherapy (MeSH)<br>#8 cancer* OR oncolog* OR neoplasm* OR kanker<br>#9 OR/7-8<br>#10 3 AND 6 AND 9<br><br>Final Search: ((MAINSUBJECT.EXACT("Dietitians") OR MAINSUBJECT.EXACT("Nutritionists") OR MAINSUBJECT.EXACT("Nutrition")) OR noft(dietitian* OR dietician* OR dietetic* OR nutrition*)) AND (MAINSUBJECT.EXACT("Asian people") OR noft(Southeast* Asia* OR South-east* Asia* OR Borneo* OR Brunei OR Cambodia* OR Indochin* OR Indo-chin* OR Indonesia* OR Lao* OR Malaysia* OR Mekong Valley OR Myanmar OR Philippin* OR Singapor* OR Thai* OR Timor-Leste OR Vietnam*)) AND ((MAINSUBJECT.EXACT("Oncology") OR MAINSUBJECT.EXACT("Cancer therapies") OR MAINSUBJECT.EXACT("Tumors") OR MAINSUBJECT.EXACT("Cancer") OR MAINSUBJECT.EXACT("Cancer surgery") OR MAINSUBJECT.EXACT("Radiation therapy") OR MAINSUBJECT.EXACT("Chemotherapy")) OR noft(cancer* OR oncolog* OR neoplasm* OR kanker))		

Table S2. Documentation of search strategy for grey literature database

Date	Database name, URL	Search strategies/words searched	# of items retrieved	# of items screened (uploaded to Endnote)
17/Aug/2023	Proquest Dissertation & Theses Global https://www.proquest.com/pqdtglobal/advanced?accountid=12528	#1 dietitians (subject heading) OR nutritionist (subject heading) OR nutrition (subject heading)<br>#2 dietitian* OR dietician* OR dietetic* OR nutrition*	36	36

Date	Database name, URL	Search strategies/words searched	# of items retrieved	# of items screened (uploaded to Endnote)
		#3 OR/1-2<br>#4 Asian people (subject heading)<br>#5 Southeast* Asia* OR South-east* Asia* OR Borneo* OR Brunei OR Cambodia* OR Indochin* OR Indo-chin* OR Indonesia* OR Lao* OR Malaysia* OR Mekong Valley OR Myanmar OR Philippin* OR Singapor* OR Thai* OR Timor-Leste OR Vietnam*<br>#6 OR/4-5<br>#7 Oncology (subject heading) OR Cancer therapies (subject heading) OR Tumors (subject heading) OR Cancer (subject heading) OR Cancer surgeries (subject heading) OR radiation therapy (subject heading) OR chemotherapy (subject heading)<br>#8 cancer* OR oncolog* OR neoplasm* OR kanker<br>#9 OR/7-8<br>#10 3 AND 6 AND 9<br><br>Final Search:<br>((MAINSUBJECT.EXACT("Dietitians") OR MAINSUBJECT.EXACT("Nutritionists") OR MAINSUBJECT.EXACT("Nutrition")) OR noft(dietitian* OR dietician* OR dietetic* OR nutrition*)) AND (MAINSUBJECT.EXACT("Asian people") OR noft(Southeast* Asia* OR South-east* Asia* OR Borneo* OR Brunei OR Cambodia* OR Indochin* OR Indo-chin* OR Indonesia* OR Lao* OR Malaysia* OR Mekong Valley OR Myanmar OR Philippin* OR Singapor* OR Thai* OR Timor-Leste OR Vietnam*)) AND ((MAINSUBJECT.EXACT("Oncology") OR MAINSUBJECT.EXACT("Cancer therapies") OR MAINSUBJECT.EXACT("Tumors") OR MAINSUBJECT.EXACT("Cancer") OR MAINSUBJECT.EXACT("Cancer surgery") OR MAINSUBJECT.EXACT("Radiation therapy") OR MAINSUBJECT.EXACT("Chemotherapy")) OR noft(cancer* OR oncolog* OR neoplasm* OR kanker))		

Table S3. Documentation of search strategy for grey literature: targeted website (nutrition association in Southeast Asia)

Date	Organisation name	URL	# of items screened
12/Sep/2023	Indonesian Nutritionist Association (Persatuan Ahli Gizi Indonesia/Persagi)	https://portal.persagi.org/	0

Date	Organisation name	URL	# of items screened
12/Sep/2023	Indonesian Dietitian Association (Asosiasi Dietisien Indonesia/AsDI)	https://ppasdi.com/	0
12/Sep/2023	Malaysia Dietetic Association	https://www.dietitians.org.my/	0
12/Sep/2023	Nutrition Society of Malaysia	https://nutriweb.org.my/	0
12/Sep/2023	Nutrition Association of Thailand	https://www.nutritionthailand.org/en/	0
12/Sep/2023	Singapore Nutrition & Dietetic Association	https://snda.org.sg/	0
12/Sep/2023	Myanmar Nutrition & Dietetic Association	https://mmnda.org/	0
12/Sep/2023	Nutritionists-Dietitians Associations of the Philippines	https://ndap.org.ph/	0
12/Sep/2023	Philippine Society of Nutritionists-dietitians	https://psnd.org.ph/	0

Table S4. Documentation of search strategy for grey literature: contact knowledge expert

Date contacted	Name of individual	# of items recommended	# of items identified for full screening
20/Jul/2023	Fitri Hudayani, S.ST., S.Gz., M.K.M., R.D.<br>Head at the central level of the Indonesian Dietitians Association (Asosiasi Dietisien Indonesia)	3	0
